# Exploring the Challenges in Covering Dental Services through Complementary Insurance in Iran: A Qualitative Study

**DOI:** 10.1155/2024/6982460

**Published:** 2024-03-11

**Authors:** Zahra Pouraskari, Reza Yazdani, Hossein Hessari

**Affiliations:** ^1^Department of Community Oral Health, School of Dentistry, Tehran University of Medical Sciences, Tehran, Iran; ^2^Research Centre for Caries Prevention, Dentistry Research Institute, Tehran University of Medical Sciences, Tehran, Iran

## Abstract

**Background:**

Financial protection is crucial for attaining universal health coverage. The inclusion of costly dental services in insurance plans poses a significant challenge for all parties involved in the insurance sector. This study aimed to investigate the challenges of covering dental services by complementary insurance in Iran during 2020–2021.

**Materials and Methods:**

This qualitative research was conducted in Iran during 2020–2021. A triangulation of methods and data sources were employed to achieve a comprehensive perspective. In-depth semistructured interviews were conducted on an individual basis, and all national documents, rules, regulations, and instructions pertaining to complementary dental insurance were thoroughly reviewed. Purposeful sampling was used to select participants from all stakeholder groups engaged in dental insurance coverage, including (1) health system policymakers, (2) insurers, (3) policyholders, (4) care providers (dentists), and (5) insured people. Six open-ended questions were formulated to explore various facets of dental insurance, including (1) development, (2) management, (3) population coverage, (4) premium calculation, (5) services coverage, and (6) payment and reimbursement mechanisms. With the consent of the participants, all interviews were recorded and transcribed verbatim. The gathered data were evaluated using a framework analysis approach in the MAXQDA20 software. Finally, the primary themes, each encompassing multiple subthemes, were identified and presented.

**Results:**

A total of 26 interviews were conducted with five groups of interviewees, and nine national documents were evaluated. Six themes were extracted, which included 18 codes from the interviews and seven codes from the documents. The extracted themes were as follows: (1) Insurance commitments and service coverage, (2) reimbursement system, (3) information system, (4) economic issues, (5) population coverage, and (6) regulation and supervision. The high cost of dental services was the most frequent challenge, followed by the insurance commitments and service coverage.

**Conclusions:**

The delivery of dental services through complementary insurance in Iran primarily faces economic and service coverage challenges. The resolution hinges on the collaboration between basic and complementary insurance sectors, the development of a unified information system for insured individuals, and the implementation of a risk-adjusted premium plan.

## 1. Introduction

Financial protection is a key element in the pursuit of Universal Health Coverage (UHC), a health objective that has been endorsed within the framework of the sustainable development goals (SDGs) [1]. Regardless of its type, health insurance serves as a financial protection, mitigating uncertainty, and financial risks associated with the healthcare costs [2].

Government-funded public health insurance enhances welfare, security, and social development. However, due to the limited scope of public insurance and the high costs of healthcare services, people often resort to the complementary insurance. Complementary insurance can supplement public insurance by either bridging the service gap through expanded service coverage or by eliminating the cost gap through increasing the extent of cost coverage.

In the majority of countries around the world, public health insurance covers only a limited range of dental services, such as emergency dental care, tooth extractions, and restorative treatments for acute pain resulting from dental caries. Other procedures, including root canal treatments, periodontal treatments, and dental prosthetics, are either not covered or necessitate copayments [3]. Despite variations in the type of services and population coverage across different countries, complementary insurance plays a pivotal role in facilitating access to the dental services [4].

### 1.1. Health Insurance in Iran

Basic health insurance encompasses a set of vital health services and goods, which are determined through prioritization and quotation processes, depending on financial, political, and social constraints. In Iran, health services, including dental services, are primarily provided by three main insurance funds: (1) the Iran Health Insurance Organization (IHIO), (2) the Social Security Organization (SSO), and (3) the Armed Forces' Medical Services Insurance Fund (AFMSIF) [5]. All basic insurance funds are required to cover the following dental services as part of the essential oral healthcare package: dental checkups, periapical/bitewing radiography, tooth extractions, surgical removal of impacted and semi-impacted teeth, supragingival scaling and oral hygiene instructions, subgingival scaling (only for individuals older than 12 years), dental prophylaxis, fissure sealant for the first and second permanent molars, and routine dental restoration for both deciduous and permanent teeth. The District Health Network (DHN) provides these services free of charge to children up to 14-year old, as well as pregnant and lactating women. For other groups, these services are offered at a 65% discount.

Currently, there are 26 complementary health insurance companies that cover comprehensive dental services. Among these companies, only one is government-owned, while the rest are privately operated [6]. Moreover, certain governmental institutions provide distinct funds, including banks, the national oil company, the national steel company, the municipality of Tehran, and the national broadcasting service known as special institutions. These institutions provide both basic and complementary health insurance to their employees and their dependents [7].

Despite the global recognition of the crucial role of complementary insurance in social welfare and health promotion, its development in Iran is still in its infancy, as evidenced by its coverage of only 20% of the Iranian population. Furthermore, while 18% of premium production is dedicated to complementary health insurance, it exhibits the highest loss ratio among all forms of complementary insurance [6].

There is a scarcity of research on the subject of complementary dental insurance in Iran. One qualitative study examined the preferences of insured households for dental insurance. This study found that an ideal dental insurance package would include both financial and nonfinancial components, such as expense reimbursement and contract providers [8]. Another study discovered that the availability of dental coverage with an appropriate benefit package encourages individuals without insurance to invest in complementary health insurance [9]. Therefore, the adequate inclusion of dental services plays a pivotal role not only in the progression of the insurance industry, but also in the promotion of oral health.

To the best of our knowledge, there has been no prior investigation into the provision of dental services by complementary insurance and the associated challenges in Iran. There is also a noticeable absence of engagement with all insurance stakeholders to consider their perspectives on this matter. Therefore, in light of this knowledge gap, in this study, we aimed to qualitatively explore the prevailing challenges of covering dental services through complementary insurance in Tehran, Iran, during 2020–2021, using a triangulation approach.

## 2. Materials and Methods

### 2.1. Study Design

In this study, we employed a qualitative approach to gather information on the prevailing challenges in covering dental services through complementary insurance in Tehran, Iran, during 2020–2021. The study was approved by the Ethics Committee of the School of Dentistry, Tehran University of Medical Sciences, (IR.TUMS.DENTISTRY.REC.1399.168). In order to gain a comprehensive understanding, we utilized a triangulation approach involving methodological and data sources triangulation. We conducted in-depth individual semi-structured interviews and explored all national documents, rules, regulations, and instructions pertaining to complementary dental insurance.

### 2.2. Data Collection

To collect data through interviews, we employed purposeful sampling to select participants from all stakeholder groups involved in the dental insurance coverage. These groups included (1) health system policymakers, (2) insurers (individuals or companies that offer insurance policies in exchange for premiums), (3) policyholders (individuals or institutions holding an insurance policy with an insurance company), (4) care providers (dentists), and (5) insured individuals (persons, groups, or organizations whose life or property is covered by an insurance policy).

### 2.3. Inclusion and Exclusion Criteria

Policymakers were selected among the members of the Supreme Health Insurance Council and the Supreme Insurance Council. Private insurers were categorized into three groups based on their market share in complementary health insurance: those with less than 5%, those with 5%–10%, and those with more than 10% [6]. As there was only one government-run complementary insurance company, it was automatically selected. Policyholders were chosen based on the size of their insured population, representing both small and large insured groups. The insurance company websites were used to select care providers. Dentists with a minimum of 5 years of experience working with complementary insurance were selected. Insured individuals were chosen from the clientele of affiliated centers who were at least 18-year old and had maintained complementary dental insurance coverage for at least 2 years. Meanwhile, policymakers lacking sufficient knowledge in the field of dentistry, insurers and policyholders without a dedicated health insurance manager, insured individuals unable to provide information about their insurance, and dentists who exclusively practiced in the government clinics were excluded from the study.

### 2.4. Interviews

Each participant received a formal letter that included a description of the study objectives, an introduction to the interviewer, and a written informed consent form. Due to the COVID-19 pandemic, both face-to-face and telephone interviews were conducted, and the participants were asked to schedule the interview at a time that suited them. The interviewer then confirmed the appointment with a phone call. The interviews were conducted by one of the authors (Z.P.), who was a female Ph.D. candidate in community oral health and had previously received training in qualitative research through participation in two workshops. To ensure the effectiveness of the interview protocol, pilot interviews were conducted with a person from each stakeholder group, and any necessary changes were made based on feedback from the other authors. All in-person interviews were conducted in the interviewees' offices, which included various governmental organizations, insurance companies, and dental offices/clinics. The interviews were conducted from December 22, 2020 to July 10, 2021.

Each interview started with a brief overview of the study objectives and the interview process for the interviewee. If the interviewee had any queries or required further information, the interviewer addressed these questions and provided the required information. Audio recording began once the interviewee was prepared to start the interview, and the investigator assured that any other persons were not present at the interview location besides participants. However, in some interviews, a third person was present with the interviewee's permission. In-depth semi-structured interviews were carried out, guided by a set of open-ended questions. These questions were designed based on the findings of a previous study [10]. Then, the questions were pretested and finalized through a pilot interview conducted with a person from each group. Six open-ended questions were asked, each focusing on a distinct aspect of dental insurance coverage: (1) development, (2) management, (3) population coverage, (4) premium determination, (5) service coverage, and (6) payment and reimbursement mechanisms (Table S1). The insured people only answered questions 3–6. In addition, the interviewees were asked to propose solutions to challenges encountered during the interviews. All interviews were recorded with the participants' consent, and the interviewer took notes from the key points. Each interview was transcribed verbatim and saved as a Microsoft Word document. Initially, all interviews were transcribed in Persian and subsequently translated into English. Data collection continued until the point of data saturation, which is defined as the stage where the ongoing analysis of the interviews yielded no new codes. All interviews adhered to the Consolidated Criteria for Reporting Qualitative Research (COREQ) checklist.

### 2.5. Validation Criteria

We employed Guba and Lincoln's four-dimensions criteria (FDC) to evaluate the validity of the interview results [11]. In terms of credibility, the interviewer built an initial rapport with the participants, typically over 1 week. We verified the interviewer's competence and expertise to conduct the interviews. The interview protocol was validated by conducting a pilot interview in each group. The interviewer took notes to guarantee comprehensive documentation of all interviews. Our research team also held frequent meetings to derive codes and themes. Additionally, we utilized triangulation approach to increase the credibility of our results. In terms of dependability, the specifics of the study protocol were shared at the beginning of the study. We meticulously documented the participants' responses during the interviews. The research team assessed the precision and consistency of the codes and themes. For confirmability, we used two triangulation approaches, including methodological and data sources. To maintain reflexivity, the investigator's perspectives, experiences, and beliefs were acknowledged and discussed in the regular team meetings. Regarding transferability, we employed a purposive sampling technique to choose participants. The interviews were conducted until data saturation was achieved in the data collection phase.

### 2.6. Assessment of Available Documents

All national documents, regulations, and instructions pertaining to the delivery of dental services via complementary insurance were searched in the electronic resources. A research team member (Z.P.) investigated the challenges identified in these data sources and cross-verified them with the challenges derived from the interviews.

### 2.7. Data Analysis

Framework analysis, a method used in qualitative research for data evaluation, was employed in this study. This analysis comprised five stages: (1) familiarization, (2) thematic framework identification, (3) indexing, (4) charting, and (5) mapping and interpretation [12]. During the familiarization process, a content summary form was developed for each interview. The preliminary thematic framework was established based on the interviews and research questions. This framework was then continually validated through repeated iterations of the familiarization process.

The initial coding was conducted by the interviewer using the MAXQDA Version 2020. To increase the validity and confirmability of the text indexing, a second researcher independently coded the interviews. This indexing process was applied to all interviews, with any discrepancies resolved through discussion. The research team rechecked all extracted codes and themes to ensure full agreement. The themes were then cross-referenced with the interviewees' perspectives based on an analysis chart. The interpretation of themes followed a process similar to indexing. Upon completion of the interview analysis, all documents, rules, regulations, and instructions were coded using MAXQDA 2020. The final codes were established in alignment with the findings from the interviews.

### 2.8. Methodological Considerations

The interviews were circulated back to the participants for validation of their authenticity. Furthermore, the Kappa agreement coefficient was measured to determine inter-rater reliability. The ultimate agreement coefficient between the two researchers was 0.95.

## 3. Results

### 3.1. Interviews

A total of 26 interviews were carried out in this study, involving five health system policymakers, five insurers, four policyholders, four dentists, six insured individuals, and two managers from special institutions. No interviews were repeated. Among the chosen stakeholders, one policyholder declined to participate in the study citing institutional privacy, and one policymaker did not respond to the researcher's invitation.

Out of the total participants, 69% were men. The mean (SD) of age was 47 (7.7) years. Eleven interviews were conducted in-person, while the remainder took place over the phone. The interview durations varied from 14 to 75 min, with an average length of 37 min. The characteristics of the interviewees are presented in Table 1.

Upon completion of the interviews, six themes and 18 codes were identified, as outlined in Table 2.

### 3.2. Documents

An initial search of electronic resources was conducted by one of the authors (Z.P.) to find documents, rules, regulations, and instructions pertaining to complementary insurance. This search yielded nine national documents. All these documents had an indirect relation to the provision of dental services via complementary insurance, with no document specifically dedicated to the dental services. While seven codes were extracted from these data sources, they did not contribute any new codes or themes to those identified from the interviews.

### 3.3. Theme I: Insurance Commitments and Service Coverage

#### 3.3.1. Low Insurance Limit for Dental Services

*(1) Policyholders and Insured People*. Policyholders and insured individuals expressed that the coverage limit for dental services was typically quite low. Consequently, despite possessing complementary insurance, insured individuals found themselves bearing the majority of the costs through out-of-pocket payments:

*“Given the high cost of dental services and the minimal reimbursement provided by insurance companies, insured individuals are required to make out-of-pocket payments, even for services that are covered by insurance.” (Interviews 19*)

*“The coverage limit for dental services under a one-year contract is relatively low.” (Interview 21)*

#### 3.3.2. Lack of Clear Principles for Risk Assessment and Premium Determination

*(1) Insured People, Policyholders, and Insurers*. All of the insured people pay the same premium rate. This premium is not based on the individual actual risk, as no initial oral examination is performed for dental coverage in complementary insurance:

*“A major concern is that individuals with varying degrees of oral disease are charged the same premium. It would be more appropriate to conduct an oral examination prior to insuring an individual.” (Interview 1*)

*“The risk of oral diseases is not first evaluated. Ideally, each insured individual should undergo an examination, and an electronic health record should be created. This would allow for the assessment of premiums based on each person's condition.” (Interview 13*)

*“One of the insurance system problems is the lack of difference between people with various oral health conditions. There is no initial examination before insurance application.” (Interview16*)

#### 3.3.3. Lack of Attention to Preventive Services

*(1) Dentists and Policymakers*. Commercial insurance companies primarily cover treatment services, with preventive services either being unavailable or extremely limited and only covered for certain age groups:

*“Dental insurance is only treatment-focused. Insurance companies lack objectives or strategies for prevention.” (Interview 4*)

*“Preventive and health-oriented services are overlooked in dental insurance. Prevention does not consider as a part of dental coverage by insurers.” (Interviews 25*)

#### 3.3.4. Low Service Quality Provided by Contracted Providers

*(1) Insurers and Policyholders*. Some insurers mentioned the low quality of services provided in contracted dental centers as a challenge resulting in dissatisfaction among insured individuals:

*“Some insured people complained about contracted centers. The low level of hygiene, use of low-quality materials, and the staff's behavior are the main factors that make the insured patients refuse to visit the contracted centers.” (Interview 13*)

According to policyholders, the quality of services provided in the contracted centers was inappropriate, which is related to low dental tariffs:

*“Contracted centers usually have poor service quality. The insurer considers low dental tariffs, and providers do not use good material to compensate for the insurer's tariffs.” (Interview 22*)

#### 3.3.5. Inadequate Contracted Providers

*(1) Policymakers, Policyholders, and Insured People*. According to some interviewees, a major challenge was the limited number of contracted providers and their uneven geographical distribution. This situation often compelled insured individuals to opt for the closest centers to their workplace or residence and pay all expenses out of pocket:

*“At times, the insured individual's residence is located too far from the contracted center, a situation that arises due to the unsuitable geographical distribution of these centers.” (Interview 2*)

*“One of the problems is the scarcity of contracted providers across all geographical regions.” (Interviews 21*)

*“The number of contracted providers is insufficient. Given my busy schedule and limited time, I often find myself visiting dental centers close to my office, most of which are not under contract.” (Interview 26*)

### 3.4. Theme II: Reimbursement System

#### 3.4.1. Inappropriate Service Authentication and Reimbursement

*(1) Insured People and Dentists*. Some insured individuals and dentists highlighted the lengthy reimbursement time from insurance companies. They identified this issue as a contributing factor to their financial losses:

*“Insurance companies tend to delay cost reimbursements. They often mention reasons, such as incomplete documentation to justify these delays. Generally, their performance in terms of reimbursement has been unsuccessful. I am dissatisfied with their reimbursement.” (Interview 24*)

All insurance companies are cautious when reviewing dental claims, aiming to minimize payouts. Employing deductions are one strategy to decrease the loss ratio. However, some dentists and insured individuals consider certain deductions to be unjustifiable:

*“Insurance companies occasionally apply deductions even when the documentation is complete. They may even exclude certain services and avoid covering their costs.” (Interview 18*)

### 3.5. Theme III: Information System

#### 3.5.1. Lack of Knowledge about Contract Details among Insured People

*(1) Insured People*. The majority of the insured individuals indicated that they lacked comprehensive information about their insurance contract. They were often only informed about the insurance limit of dental services:

*“We lack sufficient information about dental services. Our knowledge is limited to the insurance cap and the list of covered services. The specifics of the contract remain unknown to us.” (Interview 24*)

#### 3.5.2. Lack of an Integrated Information System between Insurance Companies

*(1) Dentists and Insurers*. Dentists and insurers highlighted the absence of an integrated information system as a significant challenge. This issue resulted in the delivery of redundant services to certain insured individuals, thereby causing financial losses to insurance companies:

*“In my opinion, a major challenge is the absence of communication among insurance companies. These companies lack a system for transferring independent client (patient) records between them.” (Interview 4*)

*“There is no unified record of oral health conditions, nor of the type and quantity of services provided to insured individuals. Currently, a person's information is confined to a single insurance company and is not shared among different companies.” (Interviews 13*)

### 3.6. Theme IV: Economic Issues

#### 3.6.1. High Costs of Dental Services

*(1) Policyholders, Policymakers, and Insurers*. Dental services, being among the costliest health services, were identified as a primary challenge by over half of the interviewees:

*“One of the main challenges is the high cost of dental services. As a result, insurance companies set a limit for the provision of dental services, depending on the policyholders' financial support.” (Interviews 6*)

*“Given the high cost of dental services, there is a great tendency toward complementary dental insurance. However, dental services are among the least appealing subjects for insurance companies, as they contribute to an increased loss ratio.” (Interview 16*)

*“Dental services are expensive due to their dependence on materials and equipment, as well as the high professional status of dentists.” (Interview 25*)

#### 3.6.2. Major Financial Contribution of Dental Services to Complementary Insurance

*(1) Insurers and Policymakers*. Basic insurance companies, such as the SSO and the IHIO, usually pay very low rates for dental services, resulting in an increase in the cost share of complementary insurance companies:

*“The share of dental services is less than 1% of the total cost of basic insurance, while this rate is more than 15% for commercial insurance companies.” (Interviews 6*)

*“The majority of financial burden of dental services is imposed on complementary insurance companies.” (Interview 8*)

#### 3.6.3. Actual Risk Associated with Dental Service Coverage

*(1) Policymakers and Insurers*. Some policymakers and insurers believed that most people have a poor oral health. This means that insurance companies face an actual risk rather than a potential risk in the field of dentistry:

*“The insurer covers the risk and assumes that the risk does not exist but may happen. In Iran, oral health care is not properly provided to people. Therefore, almost everyone needs dental services. This means that the insurance company encounters a realized risk.” (Interview 6*)

*“Insurance serves as a safeguard against potential risks, not events that are certain to happen. In dentistry, the occurrence of risk is a certainty, leading insurance companies to either decline coverage for dental services or impose high premiums for them.” (Interviews 25*)

### 3.7. Theme V: Population Coverage

#### 3.7.1. Lack of Dental Coverage for Small Populations

*(1) Dentists and Policyholders*. According to some dentists and policyholders, insurance companies do not cover dental services for small groups. These companies stipulate a minimum number of individuals for dental coverage based on the agreements between themselves:

*“Insurance companies usually insure groups of 500 people or more.” (Interview 4*)

*“Insurance companies typically exclude dental services for small groups, generally providing coverage for populations larger than 1,000 individuals.” (Interview 5*)

#### 3.7.2. Lack of Individual and Family Dental Insurance

*(1) Policymakers*. Policymakers identified the absence of individual and family dental insurance as a problem, arguing that many people with private or solo practices are unable to take advantage of group dental coverage:

*“Many families desire family dental insurance coverage, which is often denied by insurance companies. This lack of support results in significant financial difficulties for these families.” (Interview 2*)

### 3.8. Theme VI: Regulation and Supervision

#### 3.8.1. Dual Position of the Central Insurance Organization (CIO)

*(1) Policymakers*. Per regulations, insurance companies are required to contribute a portion of their income to the CIO. This is a significant factor contributing to the poor oversight of instruction implementation and insurer performance:

*“The Central Insurance Organization currently lacks sufficient authority. As a governing body, it is responsible for the legislation and supervision of insurance companies. Currently, this organization assumes a dual role of governance and partnership with insurance companies, given that a portion of the companies' revenue is allocated to the Central Insurance Organization.” (Interview 25*)

#### 3.8.2. Lack of Specific Complementary Dental Insurance

*(1) Policymakers*. Commercial insurance companies do not provide dental insurance independently. Instead, dental services are included as additional coverage alongside other health services:

*“Under current central insurance laws, dental services cannot be insured independently. Instead, they are included as part of the coverage for hospital, paraclinical, and other outpatient services.” (Interview 25*)

#### 3.8.3. Lack of Identical and Imperative Tariffs

*(1) Policyholders, Policymakers, Insurers, and Insured People*. Until 2020, dental tariffs were determined by the Syndicate of Insurers, which encompasses all commercial insurance companies. From 2020 onwards, the Ministry of Health began setting these tariffs. However, about one-third of interviewees felt that these tariffs were typically low, and most dentists appeared not to adhere to them:

*“The lack of a standardized dental tariff that all dentists are required to adhere to is problematic.” (Interview 6*)

*“If you visit different dental offices or clinics even in the same city, you will face different tariffs. There are no identical dental tariffs.” (Interview 8*)

*“Although the Ministry of Health and the Syndicate of Insurers have established specific tariffs for dental services, each dentist sets their own rates.” (Interviews 16*)

*“The dental tariffs set by insurance companies are significantly lower than the actual costs incurred by patients and charged by dentists.” (Interview 23*)

#### 3.8.4. Poor Supervision of Contracted Providers

*(1) Insurers*. Some insurers have highlighted the issue of inadequate oversight in the provision of dental services. This lack of stringent supervision is cited as a contributing factor to the substandard quality of services provided by affiliated providers:

*“Unfortunately, the lack of adequate oversight over contracted providers leads insured individuals to favor providers who are not under contract.” (Interview 13*)

#### 3.8.5. Fraud in Dental Insurance

*(1) Policymakers, Policyholders, and Insurers*. Nearly half of the interviewees indicated the likelihood of fraudulent practices and falsification in insurance paperwork. They expressed the belief that both insured individuals and dentists played a role in perpetuating this issue:

*“Instances of fraud have been reported in relation to dental service providers. Some dental claims pertain to services that were allegedly not provided.” (Interview 13*)

*“There is also document forgery. Unfortunately, the insured people falsify documents to use their annual dental insurance limit.” (Interview 21*)

*“The primary source of fraud in dental services stems from insured individuals, often through document forgery. Dental clinic staff may also participate in these fraudulent activities.” (Interviews 25*)

### 3.9. Solutions

We asked all participants to offer their solutions for addressing the existing challenges. The interviewees suggested some solutions to overcome the challenges of dental coverage by complementary insurance. These comprehensive solutions, offered by all participants, could enhance dental coverage for all stakeholders. These suggestions are classified into seven categories (Table 3).

## 4. Discussion

This study focused on the existing challenges in providing dental service coverage through complementary insurance in Iran. We conducted interviews with all stakeholders involved in insurance coverage and scrutinized all national documents, rules, regulations, and instructions pertinent to complementary dental insurance. This triangulation approach, enabled us to comprehensively identify the challenges and enhance the credibility of our findings.

Insurance serves as a facilitator in accessing dental services. Health systems strive to expand their dental insurance coverage, which is associated with the increased use of dental services [13]. Addressing the challenges in this field and implementing suitable solutions can play a pivotal role in attaining universal dental insurance coverage and minimizing out-of-pocket expenses. Our research developed a comprehensive framework, consisting of six main components, to scrutinize the challenges related to the inclusion of dental services in complementary insurance. The identified themes and a series of proposed solutions are discussed in the following sections.

### 4.1. Insurance Commitments and Service Coverage

Our findings underscored several challenges associated with the commitments and service coverage of insurance companies. The most significant challenges were limited insurance coverage for dental services, insufficient number of contracted providers, subpar quality of services provided by these providers, neglect of preventive services, and the absence of transparent principles for risk evaluation and premium calculation.

The current study discovered that dental services were subject to a low insurance cap, resulting in the majority of dental costs being covered out-of-pocket. Given the expensive nature of dental services and the regulations set by the CIO, this cap helps to manage the proportion of dental services within overall healthcare expenditures. While a cost limit is imposed on voluntary health insurance (VHI) in certain European nations, countries like the United Kingdom, Denmark, and the Netherlands do not have such limits [14]. In general, among all the insured healthcare services, dental services faced the most significant financial obstacles. This observation aligns with the findings of a 2016 study by Vujicic et al. [15] which indicated that, regardless of age or insurance type, insured individuals faced the greatest financial barriers when accessing dental services compared to the other healthcare services.

Our findings showed no well-defined criteria for oral health risk assessment and premium determination, inconsistent with other countries [16, 17]. In Germany, the premium for private insurance is determined based on risk, while the premium for public insurance is calculated on a community basis [18]. In addition, the private dental insurance premium is lower than statutory insurance and the service packages are flexible. However, family members must be insured separately [17]. The lack of risk-based dental insurance in Iran might be related to the absence of a comprehensive oral health assessment infrastructure.

The present study suggested that complementary insurance companies paid no attention to preventive dental services. A study conducted in 2018 discovered that students with complementary dental insurance in Iran were 6.2 times more likely to use dental services compared to those without such insurance. Interestingly, these insured students also had a higher decayed, missing, and filled teeth (DMFT) index compared to their uninsured counterparts [19]. Similarly, in most of the examined European countries, the focus of service coverage is more on treatment procedures rather than on preventive dental services [3]. Observations from various countries suggest that broadening insurance coverage to include preventive services can enhance oral health across all age groups [17].

Another issue raised by the interviewees was the substandard quality of services offered by affiliated centers. The quality of service plays a crucial role in the selection of a dental center for insured individuals [20] and is under constant scrutiny by private health insurance [21]. Choi et al. [21] demonstrated that there was a marginal improvement in the overall quality of dental care from 2015 to 2019 among children with private insurance in the United States. However, the quality score was notably lower and showed a decline among adolescents [21]. The disparity in service quality underscores the necessity for policy interventions that extend beyond merely offering dental coverage.

The interviewees also reported the issue of insufficient contracted providers. Similarly, a study by Wehby et al. [22] found that as the distance to the dentist's office increased, the usage of comprehensive dental exams decreased among children enrolled in the Medicaid program. Furthermore, McKernan et al. [23] found that 11% of enrollees in the Medicaid Dental Wellness Plan (MDWP) had unmet dental needs due to issues with transportation, one of which was the concern about the costs of transport. Suitable coverage of dental services serves as a motivating factor in the acquisition of complementary health insurance [24]. To enhance the commitments and service coverage of insurance, it is suggested to offer dental coverage with a risk-based premium, choose health centers that are evenly distributed geographically, and improve both the quality of services and the extent of cost coverage.

### 4.2. Reimbursement System

The present study indicated that the insured people and dentists were not satisfied with the reimbursement process. The interviewees believed that this process should be expedited and that insurers should refrain from making unwarranted deductions; these views align with the results of a recent local study [8]. The reimbursement process plays a crucial role in the choice of a complementary health insurance company [25]. The adoption of new technologies can minimize the need for face-to-face interactions and expedite processing times. Furthermore, the implementation of an electronic health insurance card can reduce bureaucratic procedures and administrative tasks, thereby enhancing financial transparency [26, 27].

### 4.3. Information System

An integrated information system is beneficial as it facilitates access to large data and enables the provision of personalized services to clients. However, based on the interviews, such a system is currently absent in Iran's complementary insurance market. Additionally, there are no available reports on dental plans and their specifics in the CIO Statistical Yearbook. Generally, insurance companies possess large data on their insured clients and the services they covered. Therefore, analysis of this data can significantly contribute to the growth and evolution of the commercial health insurance sector [28].

A poor understanding of the specifics of dental coverage, including the services covered, can result in dissatisfaction among insured individuals. Previous studies have reported that approximately 29%–66% of insured individuals were not aware of the dental benefit structure and services [20, 29, 30]. The adoption of advanced technologies for communication and data sharing offers multiple advantages for insurers, such as improved risk assessment and prevention, fraud detection, service innovations, reduction of moral hazards and adverse selection, and increased consumer participation in the sales process [26, 28].

### 4.4. Economic Issues

Oral diseases and dental services are costly across the world [31]. Various factors, including significant price fluctuations of dental materials and equipment over the past 3 years and international sanctions, have led to an increase in the cost of dental services in Iran, thereby influencing dental tariffs. A study highlighted that, factors such as office rent, cost of raw materials, staff salaries, disease severity, and the dentist's skill level, should be considered when determining tariffs [32]. Despite the recent growth of VHI in low- and middle-income countries, it has not led to a reduction in out-of-pocket healthcare expenditure [33]. In 2012, Ramraj et al. [34] observed a decrease in the proportion of middle-income individuals in Canada with employment-based dental insurance over the past decades. The study predicted a rise in the number of under-insured and uninsured individuals due to the escalating cost of dental insurance coupled with economic difficulties [34].

While basic insurance organizations in Iran provide coverage for over 90% of the population, dental expenses account for less than 1% of these organizations' total reimbursements. In contrast, public sources contribute to an average of 31% of dental expenditures in European countries [35]. In Iran, approximately 16% of all expenditures are dedicated to dental costs by complementary insurance companies. This is comparable to certain European countries where VHI represents a 17% share of dental expenditures [35]. Dentistry poses an actual risk for complementary insurance companies. A recent study found that a significant portion of the Iranian population has unmet dental needs [36]. As demand for dental services grows, insurers face greater risk. To lower this risk, they often choose not to provide coverage for these services.

### 4.5. Population Coverage

Our research indicated that dental services are only covered by complementary insurance when it is part of group health insurance, ideally for large groups. The latest CIO Statistical Yearbook revealed that only 20% of the population has such complementary health insurance [6]. Similarly, group insurance is the most common form of VHI in 16 European countries, while the share of individual and group insurance sales is almost equal in France and Italy [14]. In Germany, private health insurance is available only in the form of individual schemes [17]. Group policies prevent adverse selection by insured individuals, lower costs, and boost business for insurers. However, they may lead to increased inequality in access to VHI [14]. Complementary dental insurance is often a privilege associated with higher education levels, higher income, and residence in wealthier areas or the capital city [14, 37, 38]. Overall, expanding complementary dental insurance to small groups and offering family or individual coverage in addition to group policies can enhance access for vulnerable populations and promote universal health coverage.

### 4.6. Regulation and Supervision

Currently, private health insurance companies in Iran operate as commercial entities under the supervision of the CIO. In many European countries, VHI is regulated as a financial service by organizations, including the central banks and insurance regulators, typically under the Ministry of Finance. In countries where VHI covers additional user charges, regulation is either shared between financial institutions and the Ministry of Health as in Hungary and Belgium, or solely handled by the Ministry of Health or other health organizations as in Ireland and Slovakia [14]. Enhanced legislation for complementary insurance companies, in collaboration with the Ministry of Health and CIO, could be beneficial. Improvements to complementary dental insurance might be achieved by reducing the CIO's financial reliance on insurers, revising existing laws, creating more explicit rules for dental insurance, and potentially setting up a separate insurance scheme specifically for dental services.

Since 2020, the Ministry of Health in Iran has officially set dental tariffs that are significantly lower than the total cost of dental services. In contrast, many European countries determine the cost of dental services through negotiations between insurers and providers, resulting in costs that exceed public prices [14]. In Germany, private pricing for dental services is determined by the government, whereas in Denmark, insurers negotiate to achieve lower prices [14].

According to the interviewees, there is no specific entity or process overseeing the provision of dental services. While several organizations, including the Ministry of Health and various insurance organizations, are officially responsible for supervising health services in Iran, the Ministry of Health, despite its significant role, has not been sufficiently successful in this area [39].

Health insurance fraud can take various forms, including the submission of false claims, duplicate claims, and fraudulent billing for services that are not provided [40]. Insurance companies strive to detect and mitigate fraud, but their success can be hindered by legal and organizational obstacles. Fraud reduction strategies include having trusted dentists evaluate dental claims, using electronic service registration systems and data mining techniques. The most commonly used data mining techniques for fraud detection in insurance include the support vector machine (SVM), Naïve Bayes, and random forest [41].

### 4.7. Strengths and Limitations

This qualitative study used a triangulation of methods and data sources to enhance the credibility of its findings. Interviews were conducted with all stakeholders of insurance coverage, and all national documents, rules, regulations, and instructions related to the complementary dental insurance were reviewed. The selection of participants from various provinces helped improve the generalizability of the results.

Similar to any qualitative study, our findings may not apply to all beneficiaries of complementary insurance across the country. The limitations of this study include potential recall bias from insured individuals recounting past experiences, the possible influence of a third person present during some interviews, and the exclusion of individuals with past complementary dental insurance or those who declined to participate due to time constraints or objections to voice recording.

## 5. Conclusions

This study identified numerous challenges related to the coverage of dental services by complementary insurance, with most issues associated with the insurance commitments and economic issued. Economic challenges both raise the cost of dental services and coverage premiums and diminish the affordability of complementary insurance. Revision of the basic dental benefit package and increasing the financial contribution of basic insurance could help to reduce out-of-pocket expenses for vulnerable populations. Moreover, improving the quality of dental services can be used as an incentive for people to purchase complementary dental insurance. It appears that promoting cooperation between basic and complementary insurance companies in legislation and policymaking can help to tackle legal and regulatory challenges. Also, cultivating an appropriate culture of insurance usage and setting up an integrated information system can address some regulatory and information-related issues.

## Figures and Tables

**Table 1:** Characteristics of participants in individual in-depth interviews.

Characteristics		Health policymakers	Insurers	Policyholders	Dentists	Insured people	Special institution managers
Age, mean (±SD)		53.2 (±3.5)	48.8 (±6.5)	44.5 (±4.2)	46.5 (±8.8)	41.7 (±10.4)	49.0 (±5.7)

Gender	Male	5	4	3	3	2	1
	Female	0	1	1	1	4	1

Education	Bachelor or lower	1	0	3	0	3	1
	Master of science	0	1	1	0	1	0
	PhD/DDS/MD	4	4	0	4	2	1

**Table 2:** Themes and subthemes extracted from interviews and national documents.

Theme	Subtheme
Insurance commitments and service coverage	(1) Low insurance limit for dental services ^*∗*^ (2) Lack of clear principles for risk assessment and premium determination (3) Lack of attention to preventive services (4) Low service quality provided by contracted providers (5) Inadequate contracted providers

Reimbursement system	(1) Inappropriate service authentication and reimbursement

Information system	(1) Lack of knowledge about contract details among insured people (2) Lack of an integrated information system between insurance companies

Economic issues	(1) High costs of dental services ^*∗*^ (2) Major financial contribution of dental services to complementary insurance ^*∗*^ (3) Actual risk associated with dental service coverage ^*∗*^

Population coverage	(1) Lack of dental coverage for small populations (2) Lack of individual and family dental insurance ^*∗*^

Regulation and supervision	(1) Dual position of the Central Insurance Organization (CIO) (2) Lack of specific complementary dental insurance ^*∗*^ (3) Lack of identical and imperative tariffs ^*∗*^ (4) Poor supervision of contracted providers (5) Fraud in dental insurance

^*∗*^Codes derived from national insurance documents, rules, regulations, and instructions.

**Table 3:** Solutions proposed by the interviewees to overcome the challenges of dental coverage by complementary insurance in Iran.

Solution category	Proposed solutions
Premium determination	(i) Establishing premiums based on an individual's oral health status (ii) Adjusting the annual premium based on an individual's oral health performance in the previous year

Service coverage	(i) Including preventive dental services and compulsory annual checkups for all age groups (ii) Ranking clinics based on the service quality

Information system	(i) Developing an integrated information system for insured people

Economic issues	(i) Revising tariff rates for dental services in accordance with the economic condition (ii) Providing governmental support by revising the basic dental insurance package and reducing the financial burden of these services in complementary insurance

Population coverage	(i) Providing flexible complementary dental insurance based on individual characteristics, such as age and socioeconomic status to expand population coverage

Regulation	(i) Providing individual and family dental insurance in addition to group insurance (ii) Developing clear rules and revising the existing regulations and instructions

Supervision	(i) Recruiting trusted dentists to review dental claims in all insurance companies (ii) Determining a single tariff by the Ministry of Health along with strengthening the supervision of dentists (iii) Using electronic records to prevent fraud

## Data Availability

The datasets used and/or analyzed during the current study are available through the corresponding author on reasonable request.

## Abbreviations

UHC: Universal Health Coverage
SDG: Sustainable Development Goals
IHIO: Iran Health Insurance Organization
SSO: Social Security Organization
AFMSIF: Armed Forces' Medical Services Insurance Fund
DHN: District Health Network
CIO: Central Insurance Organization.

## Supplementary Materials

Supplementary Materials Supplementary materials of this manuscript include Table S1. This table consists of interview questions that were used for individual interviews. Table S1: topics and questions of semi-structured guide for interviews.
