# Supplementary material for: Exploring the Challenges in Covering Dental Services through Complementary Insurance in Iran: A Qualitative Study
Source: Int J Dent. 2024 Mar 11;2024:6982460. doi: 10.1155/2024/6982460 (PMC10948230; doi:10.1155/2024/6982460)
Supplement: Supplementary Materials — of this manuscript include Table S1. This table consists of interview questions that were used for individual interviews. Table S1: topics and questions of semi-structured guide for interviews. [file 6982460.f1.docx]

| Table S1 Topics and questions of semi-structured guide for interviews | |
| --- | --- |
| Topic | **Questions** |
| Demographic characteristics | How old are you? |
|  | What is your educational level? |
| Implementation* | “Are there any challenges on implementation of complementary dental insurance?  If yes, what do you believe these challenges are?” |
| Management * | “Are there any challenges on management of complementary dental insurance?  If yes, what do you believe these challenges are?” |
| Population coverage | “Are there any challenges on population coverage of complementary dental insurance?  If yes, what do you believe these challenges are?” |
| Premium determination | “Are there any challenges on premium determination of complementary dental insurance?  If yes, what do you believe these challenges are?” |
| Service coverage | “Are there any challenges on service coverage of complementary dental insurance?  If yes, what do you believe these challenges are?” |
| Payment and reimbursement | “Are there any challenges on payment and reimbursement of complementary dental insurance?  If yes, what do you believe these challenges are?” |

* The question was not asked the insured.
